# GHz ultrasonic sensor for ionic content with high sensitivity and localization

**DOI:** 10.1016/j.isci.2023.106907

**Published:** 2023-05-19

**Authors:** Priya S. Balasubramanian, Amit Lal

**Affiliations:** 1 School of Electrical and Computer Engineering, Cornell University, Ithaca, NY 14853, USA

**Keywords:** Physics, Applied physics, Radiation physics

## Abstract

Sensing the ionic content of a solution at high spatial and temporal resolution and sensitivity is a challenge in nanosensing. This paper describes a comprehensive investigation of the possibility of GHz ultrasound acoustic impedance sensors to sense the content of an ionic aqueous medium. At the 1.55 GHz ultrasonic frequency used in this study, the micron-scale wavelength and the decay lengths in liquid result in a highly localized sense volume with the added potential for high temporal resolution and sensitivity. The amplitude of the back reflected pulse is related to the acoustic impedance of the medium and a function of ionic species concentration of the KCl, NaCl, and CaCl_2_ solutions used in this study. A concentration sensitivity as high as 1 mM and concentration detection range of 0 to 3 M was achieved. These bulk acoustic wave pulse-echo acoustic impedance sensors can also be used to record dynamic ionic flux.

## Highlights

• A pulse-echo bulk acoustic wave GHz ultrasonic sensor for ionic content is developed
• GHz ultrasonic wavefronts allow for high spatial and temporal resolution
• The sensor is a label-free and sensitive method to detect acoustic impedance
• Both ionic content and dynamic ionic flux can be measured as a function of time

## Graphical abstract

**Figure:**
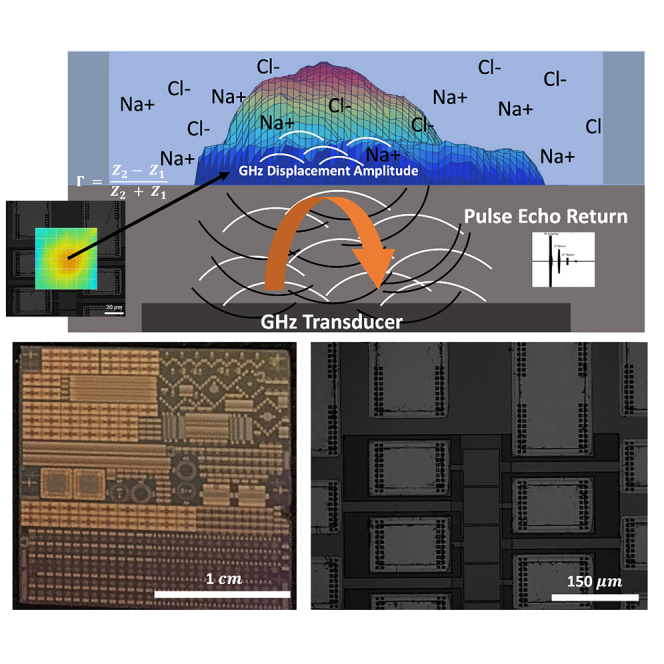

## Teaser

Ultrasound technology; Physics; Applied physics; Radiation physics; Sensor system; Devices

## Introduction

Developing high-sensitivity ionic content sensors for dynamic environments remains incredibly challenging. Rising perspectives in improving spatial resolution, temporal resolution, and detection sensitivity have become significant aspects to consider in sensor development. Contemporary sensors utilize either optical or electrical techniques, using microscale arrays or solubilized nanosensors to interrogate ionic solutions.^1^^,^^2^^,^^3^^,^^4^^,^^5^^,^^6^ However, both of these techniques suffer from electrode degradation over the long term, and potential effects such as phototoxicity can hinder the broad use of these techniques. As such, new sensing techniques for dynamic nanoscale phenomena such as ionic flux bring forth new possibilities to meet a ubiquitous need.

Sensing the nanoscale dynamics of ions in flux is important in monitoring biochemical and physiological parameters. Ionic flux is critical in signaling, conduction, and contractility in various cell and tissue lineages.^7^^,^^8^^,^^9^^,^^10^^,^^11^ Furthermore, ionic and molecular components of sweat are essential for identifying the effects of body stress.^12^^,^^13^^,^^14^ Recently, wearable sensors have been in demand to monitor sweat, and similar systems can be devised to monitor internal and external body fluids and secretions.^15^^,^^16^^,^^17^ The transport of ions is also crucial in many modern devices such as batteries, fuel cells, and the corrosion of metals. Some processing techniques, such as electroplating, depend on ionic transport and can benefit from monitoring for process optimization. Experimentally measuring the diffusion of ions in the presence of electrostatic fields can be coupled with many recent theoretical models of this process further to enhance the various downstream applications of this research.^18^

### Previous efforts in ionic sensing

While many approaches to measuring ionic behavior utilize electrical potentials, a non-electric measurement can be useful. For example, electrodes can corrode over time and require additional processing and electrical connections to the fluid. Molecular sensors suffer from the necessity to introduce a new nanoparticle into the system, and in biological applications, this leads to low lifetime due to metabolic effects and delocalization. Furthermore, several of these molecular sensors depend on photonic stimulation for readout and, as a result, introduce thermal and photonic degrading effects to the system of interest. Nonelectrical and nonmolecular interventions hold promise in sensor longevity and minimal interference with the sensing medium. Ultrasonic sensors can enable ionic measurement without having any electrodes on the sensing side.^19^^,^^20^^,^^21^^,^^22^ Furthermore, when designed to be acoustic impedance sensors, they can sense changes in medium properties with high sensitivity and minimal ultrasonic energy transmission. As such, these sensors offer minimal interference due to input energy and heightened longevity compared to electrodes and molecular sensors, which suffer from the development of capacitive layers, corrosion, molecular degradation, and delocalization through various mechanisms. While molecular sensors may have high specificity for a specific ion of interest, it is also possible to differentiate between ionic species with ultrasonic sensors using various analog and computational techniques. These techniques are surveyed with relevant work cited in the below section.

Ion-selective electrode (ISE) systems have a charge concentration nonlinearity. The well-known Nernst equation can model the ionic activity in a solution, which can be theoretically related to concentration only under certain operating principles.^5^^,^^6^^,^^23^^,^^24^^,^^25^^,^^26^^,^^27^^,^^28^ The practical limit of ionic concentration resolution is the Nernstian slope, set at 59.2 mV per decade of current density at room temperature and 61 mV/dec at body temperature. As the voltage readout from the electrode is absolute, this is the often-cited measurement sensitivity.^5^^,^^29^ This measurement is also subject to inherent electrode noise floor and signal recording limitations. Arrays of ISEs can improve the theoretical Nernstian slope response limit.^5^ Ion-selective membrane-based electrochemical transistors can accomplish super-Nernstian sensitivity at upward of 80 mV/dec, with temporal resolution at 1 s. There is still area for improvement to accomplish sub-millisecond temporal resolutions.^29^

Optical measurements of ionic content include spectroscopy-based measurements, where high sensitivity is traded for real-time measurements.^1^^,^^2^^,^^30^^,^^31^^,^^32^ Other measurement techniques involve using optical signals from dynamically binding molecular dyes, which can provide sensitivity at the nano to micromolar level and further rapid sub-millisecond response times. The optical response with the dyes can be nonlinear across the range of concentrations and potentially phototoxic, thus incompatible with long-term monitoring. Furthermore, temporal and spatial resolutions are highly influenced by background noise, nonspecific binding, and autofluorescence.^33^ While optical techniques can exhibit high dynamic range, there is usually high sensitivity to low concentration in the micromolar range but limitations to sensitivity past this. Binding kinetics can also be measured with ISEs and optical techniques using chip-scale technologies. The accuracy is subject to membrane and sensor lifetime, binding sterics, and the associated signal-to-noise.^34^^,^^35^ Furthermore, these techniques are more challenging to translate to clinical and medical settings.

Dielectric constant detection through microwave sensors is another technique that allows for high-sensitivity measurements using microscale and potentially array-based slot and metallic resonators.^3^^,^^4^^,^^36^^,^^37^^,^^38^^,^^39^^,^^40^^,^^41^^,^^42^^,^^43^ Sub-millimolar resolutions have been recorded with differential mode complementary split-ring resonator-based sensors at low concentration range.^37^^,^^44^^,^^45^ However, the resolution provided by these microwave resonators is not just dependent on the sensor design and analyte of interest but also the packaging around the sense volume. This makes these sensors susceptible to variations in packaging and boundary conditions over time. High-density, miniaturized, and channel-free microwave sensors are still in progress toward meeting the growing needs of the field of nanosensing.

Acoustic impedance sensors measure the impedance ($Z=\rho v_{ac}$), where the density and the velocity can be time-varying and include the real and the complex components.^19^^,^^22^^,^^46^^,^^47^^,^^48^^,^^49^^,^^50^^,^^51^^,^^52^ Acoustic impedance is sensitive to the density, speed of sound, and attenuation characteristics of the medium of interest. Acoustic impedance measurement sensors are useful in comparing different materials and dynamically changing material properties.^19^^,^^21^^,^^46^^,^^47^^,^^48^^,^^49^^,^^53^^,^^54^^,^^55^ Changes of 20% volume fraction and sub 100 mM concentration changes have been recorded sensitivities for these techniques.^19^^,^^21^^,^^46^^,^^47^^,^^48^^,^^49^^,^^53^^,^^54^^,^^55^ The acoustic impedance may vary due to changes in density and elasticity within the sample. Acoustic impedance sensors vary by the specific acoustic waves (SAW, bulk, shear, etc.), the operating resonance frequencies, and the method of interfacing to the sample through coupling layers. Acoustic impedance can be sensed with high resolution using analog and digital processing techniques and has various biological and material applications. The applications of acoustic impedance measurements include sensing dynamic ionic flux in biological and nonbiological media, sensing structural properties of tissue, scar tissue formation, electrolyte sensing, sensing pressure differentials, material flaw characterization, polymerization characterization, and quantification of reaction rates of microscale reactors.^56^^,^^57^

### Acoustic impedance sensors

The acoustic impedance of a lossy medium is represented as follows,

(Equation 1) Z=ρc(1−jαλ)

where *ρ* is the density of the interface medium, *α* is the attenuation parameter of the medium, *λ* is the wavelength of the acoustic wave, and *c* is the speed of sound in the medium of interest.^49^ Apart from the effect on the acoustic impedance itself, the attenuation of the medium also defines the decay length over which the acoustic wave decays exponentially. The decay length determines the sensing depth of the liquid, as the acoustic field does not penetrate significantly beyond 2–3 decay lengths. Acoustic impedance varies ideally with a density as shown in the above equation; however, there are expected deviations due to the potential dependency of α on density.

One variety of acoustic impedance sensors utilizes bulk acoustic waves (BAW sensors) with waves generated by thickness-shear or longitudinal bulk mode transducers.^56^^,^^57^ Surface acoustic waves (SAW) may be generated by interdigitated transducers and used for sensing applications.^58^^,^^59^ SAW wave penetration into surface liquids can be highly localized in-depth to an order of wavelength at the surface. SAW transducers can consume considerable surface area, and the interdigitated fingers typically are on the same side as the interdigital transducers where the liquid is to be sensed. The liquids and the electrical connections on one side can complicate the packaging as the electrical connections have to be electrically isolated from the fluidic interfaces. BAW devices can launch waves into the liquid, and the energy transmitted is governed by the transmission and reflection characteristics determined by the acoustic impedance. The transducers can be placed on the opposite side of the sensor surface. In this case, the waves travel through the substrate to the fluid-silicon interface. The BAW wave penetration in the liquid is dependent on the acoustic loss in the liquid. At kHz–MHz frequencies, the absorption depth is large, and the reflecting boundary conditions away from the solid-liquid interface typically define the depth resolution. For example, if operating at MHz frequencies, the depth of loss is tens of centimeters, and hence any package boundary condition determines the sensing volume. At GHz frequencies, the absorption depth is small, enabling a more straightforward measurement of the ultrasonic impedance without the concern of packaging determining boundary conditions. Thus, wavefront confinement is a function of the frequency both axially and laterally, defined by both diffraction limits of acoustic beams and medium attenuation properties.

### GHz ultrasonic acoustic impedance sensors – Background and operating principle

GHz ultrahigh frequency bulk acoustic waves accomplish high confinement - tens of μm for the axial resolution and down to 1 μm in lateral resolution in sense volume in aqueous medium. Furthermore, the high-frequency GHz regime allows for higher sample rates inherent in the high-frequency stimulus signal and lower in-band noise. At GHz frequencies, ultrasonic waves in water have wavelengths in the 1–5 μm in fluids and decay within 25 μm in liquids, thus lending to higher spatial resolutions than lower frequency sonic wavefronts. Given the tens of micron scale of attenuation depth, 1.5 GHz frequency scales well to a sense volume not saturated with signal solely from double-layer formation at the silicon-liquid interface. We present in this study one of the first chip-scale CMOS-compatible GHz ultrasonic acoustic impedance sensors for ionic content. There have been approaches in which GHz US has been used for mass sensing. Here, added molecules on a surface result in a change in the resonance frequency. This mass sensing technique, used in the resonant mode of the device, is not compatible with pulse transmit-receive readout.^60^^,^^61^^,^^62^ Picosecond ultrasonics has been used to investigate the propagation of ultrahigh frequency ultrasonic waves. In this approach, a laser pulse is used as a pump, and an optical probe beam can be used to measure the displacements and drive the ultrasonic motion.^63^ This method is not amenable to miniaturized, implantable, or handheld devices due to the size and cost of lasers and the space typically needed for optical beam manipulation. Furthermore, it is more challenging to configure in a flexible array format that lends more effectively to sensing systems. In addition, compared to high-sensitivity microwave resonator-based sensors, GHz ultrasonic chip-scale transmit-receive sensors used in this study for acoustic impedance measurements do not require restricted chambers for the interface with the analyte. CMOS-integrated GHz transducers can create compact devices, enabling low cost and miniature implementation.^64^^,^^65^^,^^66^^,^^67^^,^^68^ Furthermore, this technique is label-free, and does not require surface functionalization to capture the analyte of interest as it simply relies on the acoustic impedance measurement. Representative of this technology, this paper reports measurements with GHz ultrasonic bulk waves generated by a 70 *μm* square aluminum nitride, or AlN thin-film transducers fabricated on a silicon wafer. The wavefronts generated by the transducers on one side travel through the silicon substrate and undergo a reflection from the opposing surface, which is in contact with a liquid medium of interest. The wave packet reflects and travels through the silicon substrate, and is then sensed by the same transducer that actuated it. The sensed wave packet intensity is proportional to the ultrasonic reflection coefficient, attenuation and diffraction losses within the silicon transmission medium, and the electromechanical coupling coefficient. The reduction in the reflection coefficient is effectively a result of the amount of ultrasonic wave energy that entered the specimen, which is directly related to ionic content and species. The reflection coefficient between a pulse arriving from material 1 (silicon) into material 2 (liquid) is

(Equation 2) $$\Gamma =\frac{Z_{2}-Z_{1}}{Z_{2}+Z_{1}}$$

where Z_1_ is the impedance of the silicon substrate, while Z_2_ is the impedance of the fluidic medium of interest. Due to the GHz frequency of the ultrasonic waves, the maximum resolution is on the order of a wavelength in the medium of interest using a phased array operation. The carrier frequency of 1.5 GHz has approximately 9 and 1 μm wavelength in silicon and water. In this work, the beam width defines the lateral resolution attainable as a function of the ultrasonic carrier frequency. Furthermore, GHz wave packets decay swiftly in the axial direction. The attenuation is governed by an exponential drop-off in signal intensity as

(Equation 3) $$I=I_{0}e^{-\alpha \Delta x}$$

where $I_{0}$ is the intensity at the silicon/water interface and α is a frequency-dependent loss factor. The following power law may approximate the attenuation dependence on frequency,

(Equation 4) $$\alpha \left(f\right)=\alpha_{0}+\alpha_{1}{\left|f\right|}^{\gamma }$$

where *α*_0_ is often set to 0, and 0 *< γ ≤* 2. These parameters are measured experimentally, and the expression derived following the theory of time causal Hooke’s Law under power law absorption behavior of the wave-medium interaction.^69^^,^^70^^,^^71^ As described in the study by Szabo and Wu,^71^ this model is valid for frequencies up to 10^12^ Hz. For GHz frequencies, acoustic attenuation is approximated in aqueous media, as used in this paper, to be 0.134 $\frac{dB}{\mu m\;GHz^{2}}$ and is quadratically related to frequency for the frequency range of interest. At 1.55 GHz, the decay length for 3-decibel loss length is 10.7 microns. This provides an axial localization within tens of microns. As a point of reference, the use of BAW acoustic impedance sensors for sensing changes in ionic content has most often cited a tested resolution of 1%–5% by weight in minimum, which translates to 100s of mM rather than 1 mM in resolution tested.^35^^,^^72^^,^^73^^,^^74^^,^^75^^,^^76^^,^^77^^,^^78^^,^^79^ Thus, this study cites sensitivities that far exceed most all reported in the literature for a similar sensing modality.

This study shows that using the principles of acoustic impedance sensing, as governed by Equations 1 and 2, one can sense the temporal activities of dynamically changing medium in addition to the static counterparts through GHz bulk acoustic wave transmit-receive reflectometry. The thickness mode ultrasonic transmit-receive transducer operating at GHz frequencies provides high localization of sensing and sensitivity. The advantage of higher frequency ultrasonics is improved temporal resolution and heightened lateral and axial resolutions. This paper is the first study to use a chip-integrated array of GHz ultrasound transducers that can sense aqueous, time-varying media.

This study describes the techniques used to characterize and operate the transducer. The experimental design was used to test the transducer’s sensitivity to concentration in both static and dynamic settings. Information regarding sensor spatial resolution is detailed. The results describe the sensitivity to detection for static and dynamic concentrations and further the influence of heat on dissolution.

## Results

### Transducer design and characterization

Devices were fabricated at the Institute of Microelectronics, IME A∗STAR, as part of the I-ARPA TIC program. The transducers used in this study are identical to previously published devices.^66^^,^^80^^,^^81^ Identically fabricated and aligned transducers are on both sides of the 750 micron thick silicon wafer. Like experimental setups in the study by Kuo et al., Abdelmejeed et al., and Balasubramanian et al.,^66^^,^^68^^,^^80^^,^^81^^,^^82^ the identically fabricated surfaces allow for proper alignment of medium and transducer, especially in the experiments of salt dissolution described in the following sections.

**Figure 1:**
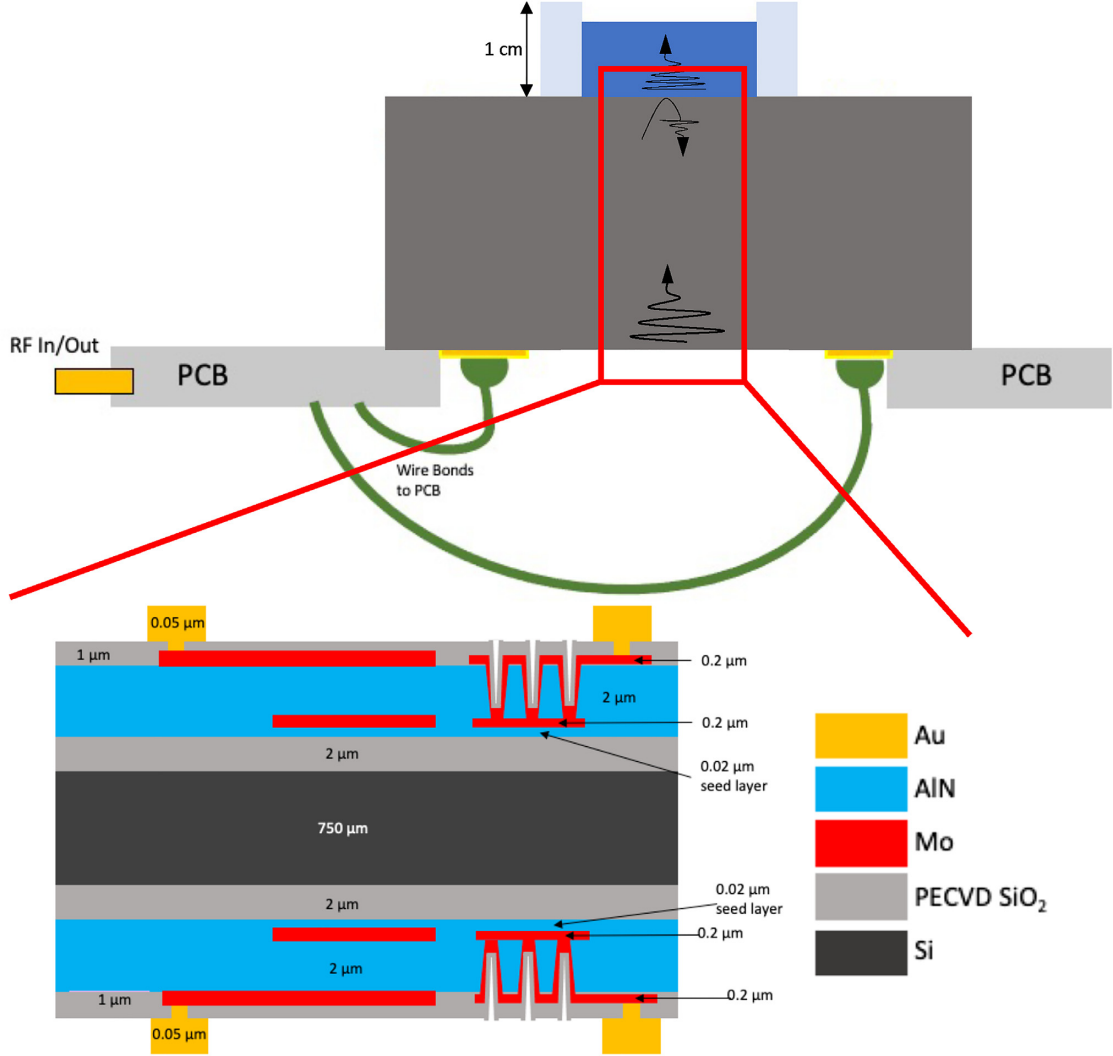
Device cross-section Device rationale and layout with the liquid medium is shown.

**Figure 2:**
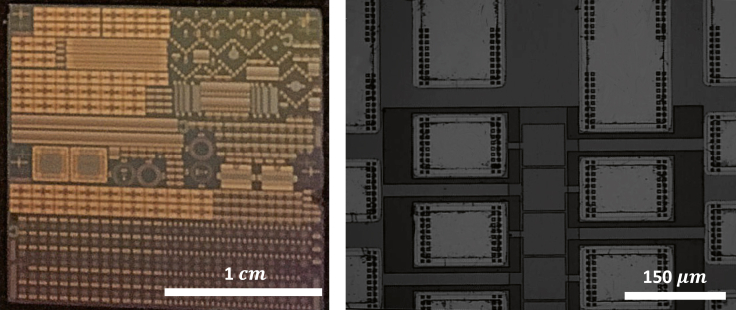
Device image Chip-scale device used for sensing photographed both at visible scale lengths (left) and high resolution through reflected light microscopy (right).

The device layout and design is detailed in Figure 1. Both surfaces are identically aligned and fabricated to allow for the alignment of the transmit-receive pulse. In addition to the PECVD-deposited SiO_2_, the thickness mode resonance of the AlN films lends to a resonance frequency with an approximate center frequency of 1.5 GHz. For the sensing experiments, the first echo’s return signal is of primary interest in measuring the medium’s ultrasonic properties. The magnitude of the first echo return signal is used for the analysis. The chip-scale device is depicted in Figure 2, at two different magnifications.

**Figure 3:**
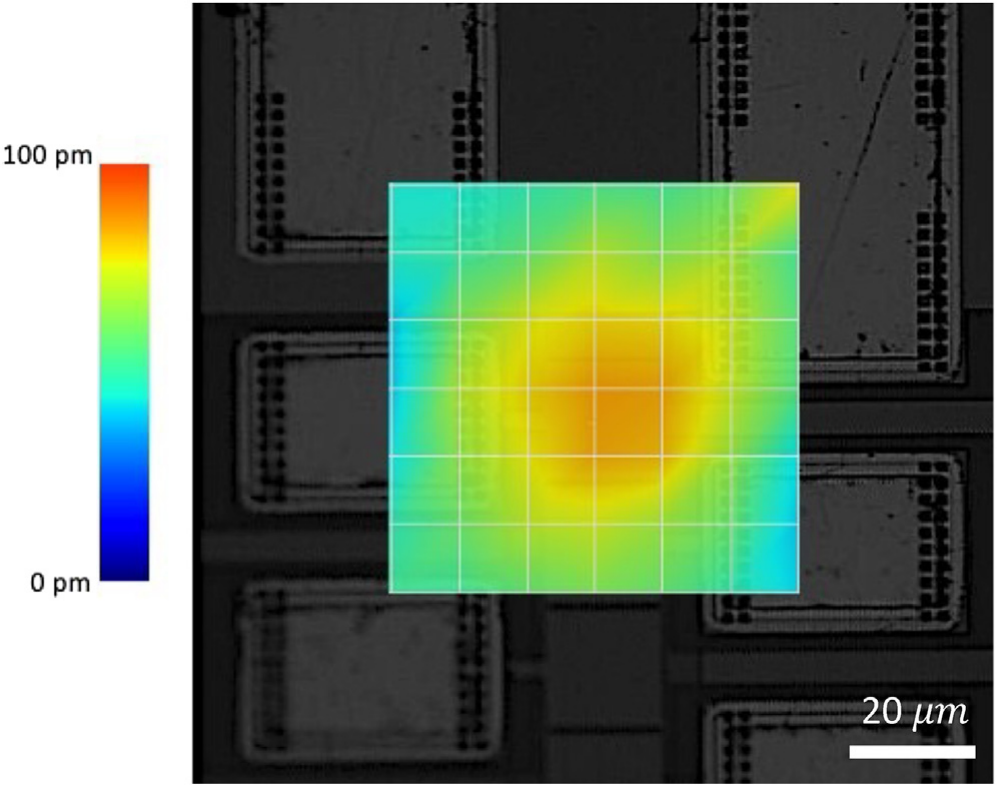
Transducer characterization for a 70 *μm* square AlN transducer The diffraction pattern obtained from Polytec UHF-120 scanning of the amplitude excited on the backside is shown.

Transducer motion in air is measured using a Polytec UHF-120 and a topical motion scan over the backside displays a diffraction pattern, as shown in Figure 3.

### Frequency response of transducer

**Figure 4:**
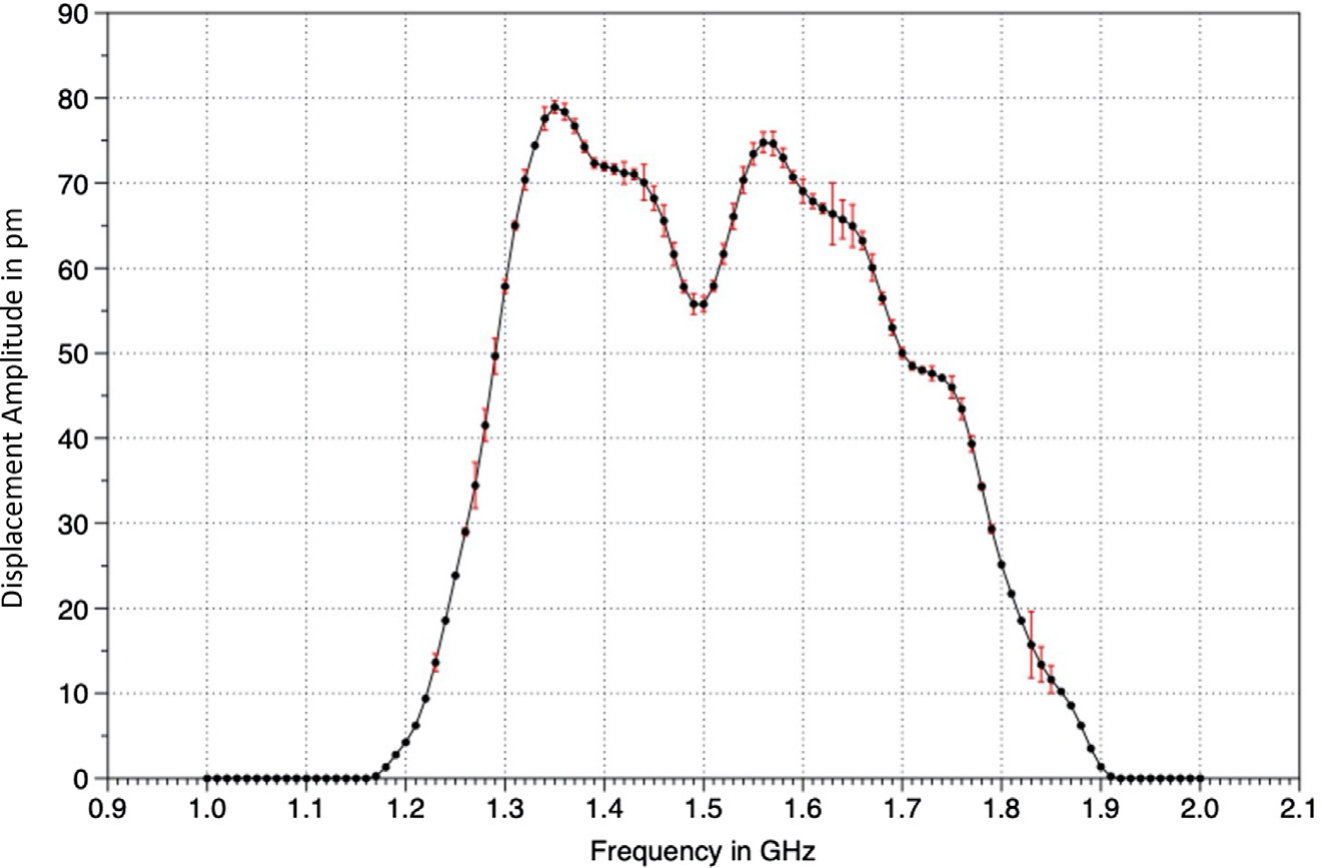
Frequency response of device A graphical depiction of the displacement amplitude across the frequency range of interest of the transducer is shown. Error bars depict standard deviation.

Data collected from the Polytec UHF interferometer under continuous-wave input to the transducer using input single-frequency waves swept across the depicted frequency range, and the transducer’s pulse-echo response using the first echo signal is shown in Figure 4. The loss of energy into the silicon substrate through diffraction lowers the resonator’s quality factor, leading to a response over a wider bandwidth. A 2 *μm* thickness AlN results in a thickness mode resonance, which could be seen in the KLM model as an open circuit resonance frequency defined by $f_{0}=\nu_{s_{ALN}}/2t_{ALN}$, where $t_{ALN}$ is the thickness of the AlN transducer. Models of the added load impedance of the silicon backing, molybdenum electrode, and oxide layers lower the actual resonance frequency and the quality factor, increasing the effective bandwidth of the transducer. The actual frequency response is complex, and its full description is beyond the scope of this paper. A more accurate acquisition of frequency response would require an automatic electrical impedance matching network on an integrated chip, including the GHz transducer, as the electric impedance of the device varies as a function of frequency. Several example systems are available in the literature and are needed to provide the most accurate device characterizations.^22^^,^^52^^,^^83^^,^^84^ In this paper, given that the operating frequency range is broad, an optimal sense frequency was chosen based on maximizing the pulse-echo return for operation.

### Analog signal acquisition of ultrasonic reflections

The signal processed and used to collect information on the acoustic impedance of the medium is the first echo of the pulsed transmit-receive echoes. The echoes are shown in Figure S1. Since this echo represents a discontinuous signal and the amplitude of the echo is what is related to the acoustic impedance of the medium, signal processing in the analog domain was employed to get heightened sensitivity and continuous sensing of the medium of interest by extracting the amplitude of the first echo.

**Figure 5:**
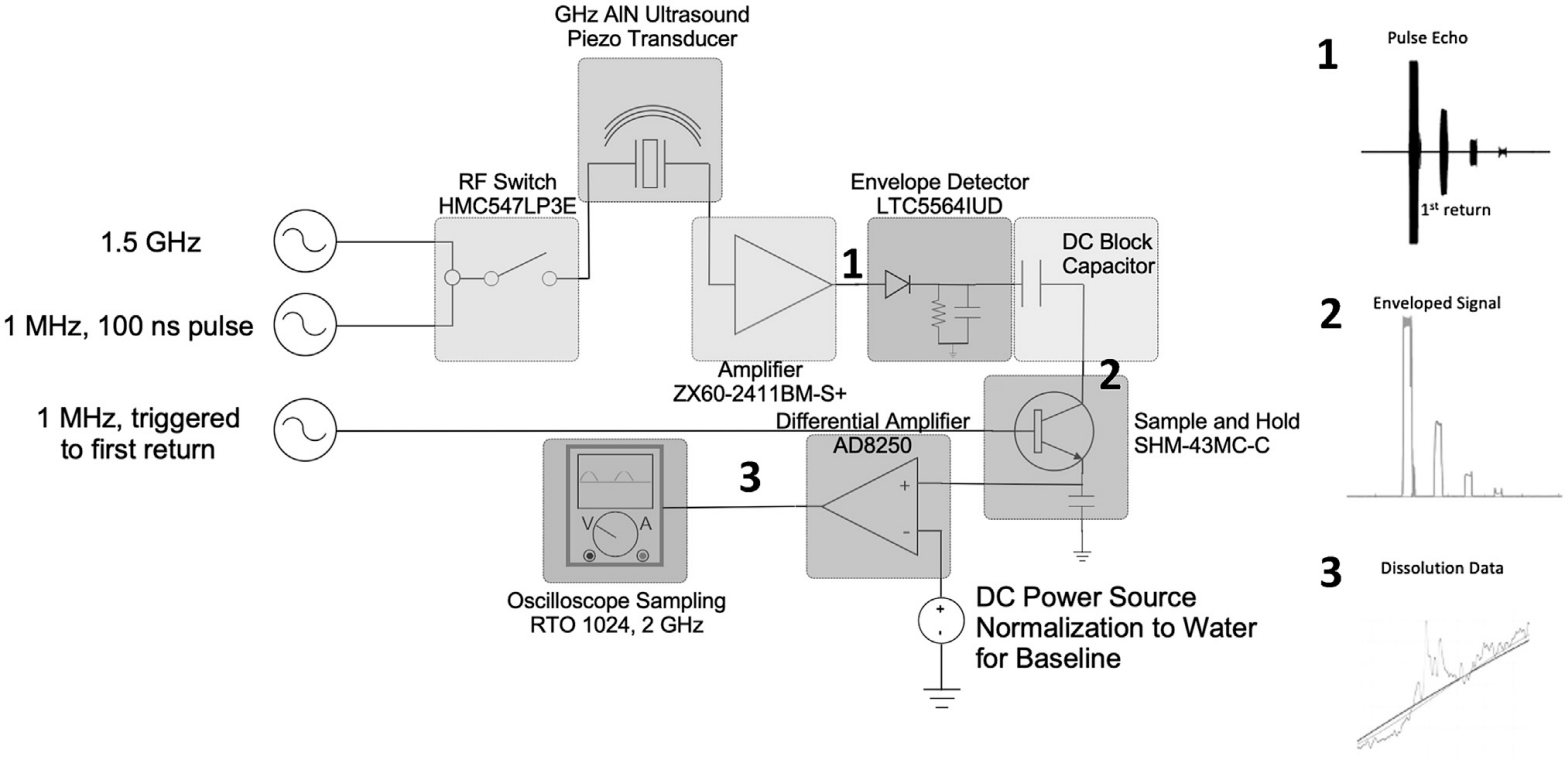
Analog processing of signal The circuit diagram used to process the signal in the analog domain prior to sampling and storing data in the oscilloscope is depicted, with each component outlined and identified.

The analog processing scheme is depicted in Figure 5. A qualitative description of the signal trajectory through the analog processing technique is described in this paragraph. The RF switch allows the continuous wave GHz signal to be sent to the transducer input using a pulse mode at 1 MHz. The signal is then returned from the transducer as a pulse-echo return and amplified using the next amplifier. This increases the signal-to-noise ratio and brings the output to the range of voltages for the envelope detector. The envelope detector generates an envelope of the RF signal through a low-pass filter. The sample-and-hold circuitry obtains the value of the signal at the first return amplitude where the signal is triggered. This signal value is used to charge a capacitor, which then holds this value through a trigger signal for a given amount of time until the next pulse is triggered. The output is then fed into a differential amplifier, which is referenced to a control threshold voltage, set to be the signal value for the reference aqueous medium with no ionic content supplemented, as shown in Figure 5. The final box is the sampling from the oscilloscope of the signal post-amplification, which shows the voltage related to the concentration through the acoustic impedance of the medium of interest. More details are provided in the STAR Methods section.

### Proposed mechanism of sensing

This sensor operates through the use of a pulse-echo signal that is generated by the backside transducer, travels through the silicon as shown in Figure 1, and then hits the chip-liquid boundary, where the wave partially transmits and partially reflects. The amplitude of the reflected signal is then sensed by the same transmit transducer, with the first echo used to preserve high signal-to-noise. Alternative configurations, including a different transmit and receive transducer, are possible, with continuous wave and pulse mode operation. The amplitude of the returned signal is related to the transmission-reflection coefficient, and thus related to the analyte properties such as density and concentration. As such, we can predict changing analyte properties with changes to the amplitude of the return signal. The raw pulse-echo signal is shown in Figure S1.

### Input electrical impedance change due to interface medium is not a substantial contribution

The material on the transducer’s backside is that air, water, or salt solution might change the effective electrical impedance for the AlN transducer or change the frequency response. The conductivity of the silicon wafer separating the front and backside of the chip could allow for the backing to influence the transducer resonance and frequency response. The first and second return experiments in the preceding section have differing sensitivities. The second return signal having a heightened sensitivity suggests that the transmission of the acoustic wave into the interface medium is dominant in the signal changes observed. However, as one cannot rule out the potential of the frequency response itself being altered or possible diffraction-associated power loss as the wave packet travels through the silicon, the RF coupled input signal was also tested and compared for air backed, deionized water backed, and 3 M NaCl backed GHz ultrasonic arrays. The RF coupled signal amplitude was 136.26 ± 10.13 mV, 136.78 ± 10.01 mV, and 135.95 ± 10.01 mV, respectively, with no statistically significant difference. Thus, it is assumed that the impedance is not varying greatly as a function of the interface medium. In the future, this phenomenon can be characterized precisely by electrical impedance matching networks.

### High concentration range ionic sensing

**Figure 6:**
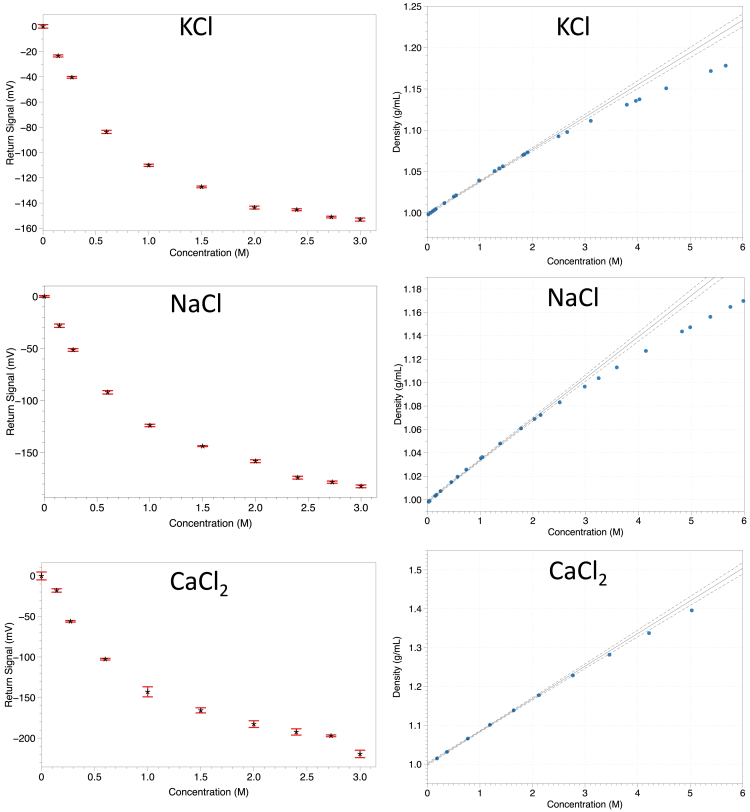
Concentration range testing The concentration of ionic solution for three different ionic species is tested across a broad concentration range up to values close to the room temperature solubility limit. Error bars depict standard deviation. Data from transducers shown on the left, and theoretical data for density and concentration relationship shown on the right from cited research.^77^^,^^85^^,^^86^^,^^87^

The concentrations tested span an extensive range, almost to the solubility limit in deionized water, from 0 M to up to 3 M. The average return signal and deviation is recorded across concentrations in Figure 6 with concentrations resolvable as plotted in the figure. Calling on the relationship expressed in Equations 1 and 2, there are still substantial deviations from the theory to be considered. This can be attributed to the following factors. First, there is a formation of an ionic layer at the surface interface of the chip and solution, which substantially complicates the calculation of acoustic impedance and also creates concentration dependencies of the α term. Furthermore, as shown in Figure 6 in the theoretical data and the right side of the figure, the relationship between concentration and density is not as expected across the entire range of concentrations.^77^^,^^85^^,^^86^^,^^87^ These effects, particularly the ionic layer formation, will substantially influence the system behavior and attributed to the observed curves shown in Figure 6. Of these quantities that have been investigated in the literature most extensively is the nonlinearity of the density and concentration relationship.^49^^,^^85^^,^^86^ In Figure 5, the subpanels to the figure’s right depict the density and concentration relationship for the entire concentration range for the ionic species of interest. Notably, the saturation of density at higher concentrations follows with the results of the acoustic impedance sensor, which is further compounded by the other nonlinear effects described previously. Various features such as theoretical deviations of the sensing medium and lower signal-to-noise due to with more acoustic power transmission into the sensing medium will influence the signal-to-noise across concentration values. Furthermore, it is shown in the literature that the acoustic impedance of an aqueous-solid interface is a function of the distance from the interface, as the density and solvent properties vary due to interfacial interactions. This effect is also characterized in the study by Mante et al. (2014) for hydrophilic surfaces.^65^ There is an expectation that surfaces ranging from highly hydrophobic to highly hydrophilic will have varying changes in the apparent density and acoustic impedance of solvent at the interface of interest.

### Concentration sensing at 1 mM sensitivity

**Figure 7:**
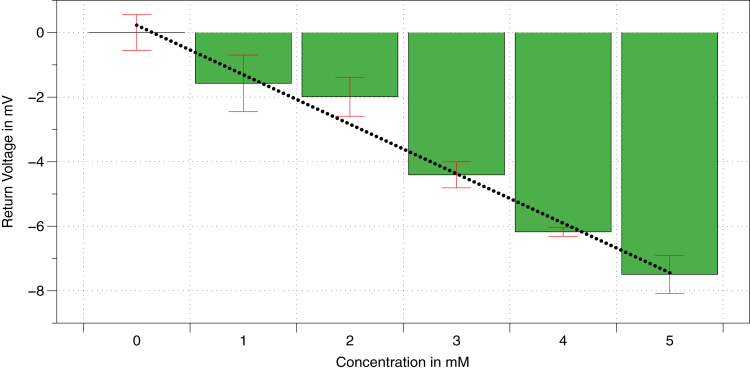
Sensor sensitivity characterization Transducer return signal versus ionic concentration of NaCl at 1-mM resolution. Error bars depict standard deviation.

Figure 7 shows sodium chloride is prepared at various concentrations ranging from 0 to 5 mM, using a calibrated high-precision small volume pipettor. The resulting curve is shown in Figure 7. A 1 mM resolution is achievable across most of the range, at a significance level of *α* = 0.05. Five acquisitions were recorded per concentration, with at least 200 returns averaged per acquisition. Standard deviation is depicted as error bars in the Figure 1. A 1 mM sensitivity is a significant landmark for ionic resolution sensing that we aim to utilize for biological and other sensing applications.^19^^,^^88^ This was achieved here across most of the samples, though it remains to be determined whether the large-range concentration sensing will maintain the resolution throughout. At the saturation level of 3M for NaCl, data suggest that a 10 mM concentration sensitivity is achievable; further research will provide details and improvements to the signal processing to obtain higher resolution.

### Ionic compound dissolution rate

Sensing the rate of change of concentration is introduced to show that a reasonable temporal resolution may be achieved with time course data that matches the expected theoretical behavior of the system. The rate of solubility, or dissolution rate, is given theoretically by $\frac{dC}{dt}=\frac{DA}{LV}\left(C_{S}-C\right)$, where C is concentration, *C*_*s*_ is solution saturation concentration, V is volume, A is the surface area of solute, L is length or radius to the measurement point, and D is diffusion coefficient.^89^ The solution for this equation is $C=C_{s}\left(1-e^{-\frac{DA}{LV}t}\right)\text{.}$

**Figure 8:**
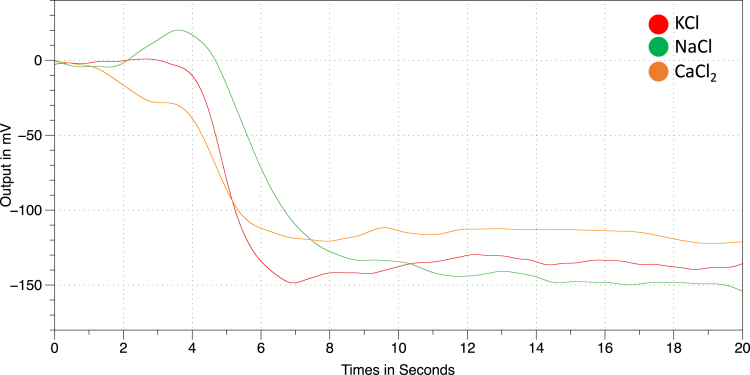
Dissolution sensing Ionic solute dissolution in aqueous solution (deionizied water) is tested and recorded for three different ionic compounds.

While model fitting poses added complexities due to the voltage-concentration nonlinearities that are beyond the scope of this study, the theoretical dissolution properties qualitatively agree with the results obtained by the sensing system. The ionic salts used were powdered into fine granules before addition at once into 100 *μL* deionized water. However, differences in the addition of salt will influence A and thus the dissolution rate. The salt introduction was performed at the same spatial location, which is 0.5 cm from the active transducer center displacement on the backside of the chip after the salt is ground to small particulates using a mortar and pestle. The introduction of the salt into the solvent was immediate and performed using a 6.3-inch tapered edge scoop-type chemical-grade spatula, gated by a flat end, tapered spatula, and the salt is released within the solvent with an immediate release of the gated spatula. The results of the dissolution of each of the ionic solutes tested are shown in Figure 8, for a total of 20 s. Further time points from data not shown suggest that a steady state is accomplished following the first 10 s of solute introduction.

### Effect of temperature on dissolution rate

**Figure 9:**
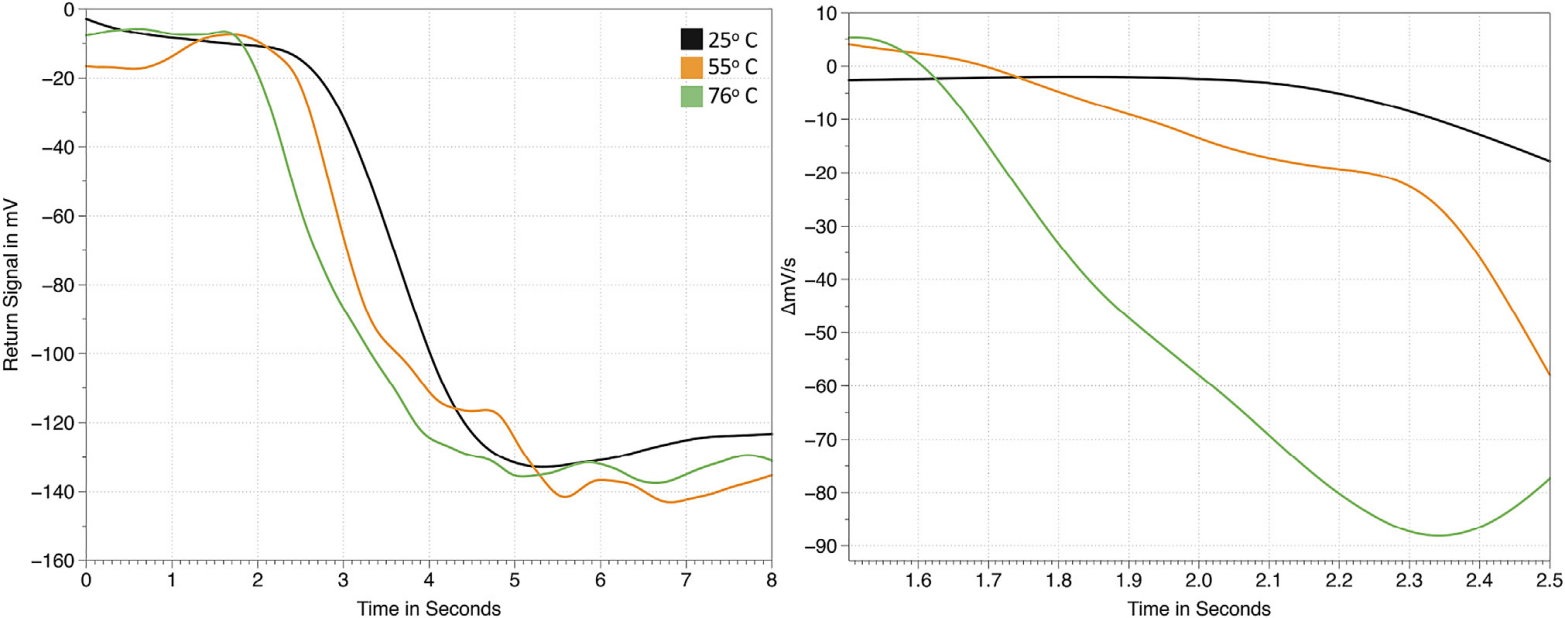
Sensing dissolution at varying temperatures A thermal heating element is used to interface with the transducer, changes in temperature of the sample volume are induced. Dissolution rate is changed for NaCl in water (left) with the rate of change of each curve shown for select time points (right) over a limited time range.

To further illustrate the sensing capacity of this system, the transducer is contacted with a thermal element that heats the sample and is calibrated with a thermocouple. Three different temperatures were tested, room temperature 24°C, 55°C, and 76°C. The dissolution time course is plotted in Figure 9. The gradient of the dissolution rate is shown for a part of the process in Figure 9 in the right subplot. It can be seen that, as the temperature is increased, the dissolution rate is also increased through the influence of the dissolution coefficient and saturation concentration (solubility) changing as a result of higher temperatures of the solution. To ascertain that heating is not causing evaporation that will lead to concentration changes in the sample volume, the highest temperature assessed in the experimental section is applied for 100 s to both deionized water and NaCl solution. Neither sample experienced significant changes in the signal return. Thus, when both deionized water and ionic solutions are subject to 76°C heating, there is no change in signal return, indicating that evaporation is not a problem within the time frame data are acquired.

## Discussion

This study is the first comprehensive characterization of chip-scale GHz ultrasonic acoustic impedance sensors used to understand and sense constant and time-varying properties of ionic aqueous solutions. Furthermore, it is one of the first BAW pulse-echo ultrasonic impedance sensors to detect dynamic ionic flux, in addition with one of the highest reported sensitivities at 1 mM. The use of BAW acoustic impedance sensors for ionic content sensing has concentration sensitivities ranging between 1% and 5%, which translates to 100s of mM depending on the particular salt of interest.^35^^,^^72^^,^^73^^,^^74^^,^^75^^,^^76^^,^^77^^,^^78^^,^^79^ Our current results not only sense dynamic flux but also report a record resolution of 1 mM, one of the lowest reported sensitivities in the literature. The nonlinearity across the concentration range of the pulse return signal is derived from the medium properties and the solid-liquid interface.^85^^,^^86^ Furthermore, this speaks for the potential non-homogeneity of the concentration gradient from the surface of the chip outward, primarily well characterized in studies regarding charges, gradients, and ion distributions of solutions at the solid-liquid interface.^65^ Evidence is shown that the nonlinearity can be at least partly attributed to the saturation of the density-concentration relation at high concentrations from theoretical values in Figure 6 (right side panels). This fascinating phenomenon can be better characterized with ionic solutions once a broad range of transducers with differing ranges of resonance frequency are developed to distinguish the effects of density-attenuation-concentration and surface interface alterations in addition to ionic double layers in deviations of acoustic impedance measures from theoretical models. Furthermore, the shear stress on the surface and vortical mixing could disrupt structured interface effects and allow for the decoupling of the system nonlinearity from the spatial inhomogeneity that could be contributing to this phenomenon. Attenuation characterization will be critical in the endeavor to fit results to a theoretical model. There is also the potential involvement of the medium influencing the wavefront itself, which could alter the wave diffraction and interference, thus leading to alterations in the response that could induce further unexpected nonlinearities. Other computational models, experimental evidence, and theoretical simulations will provide more information toward deciphering the nonlinear effects. Noise propagation techniques will allow for the derivation of signal-to-noise as a function of concentration and analog processing components. Notably, the analog processing and system itself could benefit from alternative setups, such as interferometry, through a phase-locked secondary transducer or IQ demodulation setup as in the study by Abdelmejeed et al.^64^ It is also worthwhile to note that the accompanying analog processing could be utilized in any differential mode power sensing application. We utilize the amplitude of the first return with respect to a DC reference in differential mode, but this can also be extended to use in any differential mode amplitude-based signal sensing modality. This technique requires either power, amplitude, or a similar quantity to be derived from the original signal rather than operating on a differential mode on the actual resonance frequency of the signal. This method is more subject to error as a result of phase noise and jitter, especially in the GHz regime.

This study is an exciting insight into the scaling and physics of GHz ultrasound and its interactions with ionic solutions. It also has many biosensing and chemical engineering applications, ranging from measuring single-cell and ion channel ionic flux to microreactor engineering, to materials characterization. This paper’s results can guide the development of compact CMOS-integrated GHz ultrasonic devices for applications in wearables, batteries, and new biochemical experiments where electrodes directly into the liquid are not possible. The unique localization of GHz ultrasound, as seen in Figure 3, allows for a spatial resolution unmet by other lower frequency ultrasonic devices. Furthermore, the GHz frequency itself lends to a high temporal resolution capability. This study provides insight into the next generation of acoustic sensors.

### Limitations of the study

One setback of the dissolution experiments is the inability to introduce the ionic solid to the solvent as a delta function in time. In experimental settings, the solute introduction is not instantaneous, and the rate of addition is challenging to control precisely. Microscale release reservoirs are needed for future model-based evaluation, which will be a fabrication challenge given the need to compound electronics and circuitry necessitated for controlled release in addition to the ultrasonic input on one chip. Furthermore, the surface area of the introduced solute is challenging to control and ascertain. Precision delivery and characterizations of solutes using microfluidics and automated XYZ stages can be developed for future experiments. These modifications will also inform future thermal experiments to investigate the thermal effects on the dissolution of these ionic solutes in an aqueous solution. Another potential area of improvement is in the analog processing circuitry. There are deviations in the return signal across concentrations and for each concentration tested due to the drift of DC reference sources into the differential amplifier and jitter from other pulse and trigger signals that influence gain, stability, and variability in results. This can be improved through modulation or interferometry-based techniques that allow for more accuracy and stability. Furthermore, integrated circuitry and sensor layouts of these chip-scale devices will significantly enhance signal-to-noise.

### Conclusions

This study presents the measurements of BAW GHz ultrasound pulse-echo amplitude changes to sense ionic concentration changes in a liquid medium on the sensor surface. Ionic content sensitivity down to 1 mM is achievable with this system, along with a lateral spatial resolution of less than 50 *μm* and axial resolution of less than 30 *μm*, limited by the diffraction of the transducer through silicon and the design of the transducer. Further design changes, beam steering, and focusing will improve the lateral resolution to the plausible 1 *μm* diffraction limit. Temporal resolution necessary to detect the dissolution of ionic species and the changes in dissolution rate due to temperature increase is achievable under the current system design. A 1 mM sensitivity is recorded for this BAW pulse-echo ultrasonic acoustic impedance sensor format. Future directions include automated analog circuit-based pulse-echo processing and fluid sampling device fabrication by integrating the transducers with CMOS as in the study by Abdelmejeed et al.,^68^ theory validation of GHz ultrasonic attenuation across different medium properties, and further understanding of sensing nonlinearities and system deviations. Applications include sensing dynamic ionic flux as relevant to neural action potential, transport in tissue and vascular flow, biomedical sensor development, materials characterization, and chemical reaction sensing.

## STAR★Methods

### Key resources table

**Table:** 

REAGENT or RESOURCE	SOURCE	IDENTIFIER
**Software and algorithms**		

Polytec PSV and ScanViewer	Polytec GmbH	https://www.polytec.com/us/vibrometry/products/software
MATLAB R2021b	Mathworks	https://www.mathworks.com/products/matlab.html

**Other**		

Polytec UHF-120	Polytec GmbH	https://www.polytec.com/us

### Resource availability

#### Lead contact

Further information and requests for resources and reagents should be directed to and will be fulfilled by the lead contact, Priya S. Balasubramanian (psb79@cornell.edu).

#### Materials availability

This study did not generate new unique reagents.

### Method details

For the characterization of the transducer, including frequency response, this paragraph describes the detailed methods used. This displacement data was collected from 1 to 2 GHz at frequency interval steps of 0.01 GHz to validate the transducer resonance frequency. The resonance was characterized by obtaining the displacement at the backside of the transducer when driven by a continuous wave RF signal. The displacement was sampled at a 22 *μm* diameter of the displacement maxima. Each sample within the area was averaged over ten pulse-echo received amplitudes. The raw data of the frequency response was denoised with a second-order LOESS fit that uses a moving window that is 0.06 times the data size. This frequency response is depicted in Figure 4.

For the analog processing, this paragraph describes the methods used in detail. Figure 5 depicts the analog processing electronics used to sense and amplify the amplitude of the first echo amplitude over time. The first return is commonly used as it has the largest amplitude. In contrast, the following echo returns will have signal loss from attenuation, reflection-transmission, and diffraction, though potentially more sensitivity to reflection transmission due to multiple traversals of the wave packet through the silicon cavity. This phenomenon is depicted in Figure S2, where the sensitivity of the signal return to sensing concentration for the first and second echo return as a result of acoustic impedance change is graphed. Given that the amplitude of the first echo is the largest, this study’s interest is to obtain the acoustic impedance-dependent first return amplitude, as governed by Equations 1 and 2. To obtain this value and allow for high-resolution sensing of the first return amplitude, tracking and amplification of the signal was employed. The signal from the transducer was amplified (ZX60- 2411BM-S+) and rectified, and the envelope of the movement was extracted (LTC5564IJD). Any DC charge or voltage held by the transducer was removed through a DC block high-pass filter and using a phase-matching pulse, the first echo envelope peak was tracked through a sample-and-hold circuit (SHM-43MC-C). This output was fed through a differential amplifier (AD8250). The second input to this amplifier was a DC signal referenced to the value of the analog return signal obtained for a chosen reference liquid. The reference liquid was chemical-grade deionized water for the sensing experiments in the following sections. The output of the differential amplifier was fed through an analog low pass filter (EF110) and sampled by the RTO-1024 10 GS/s oscilloscope. The signal was processed post-acquisition digitally with a local moving weighted regression across 1% of the total sample size for dynamic sensing outputs and averaged across multiple repeat trials for steady-state sensing across the acquisition window.

For the ionic content sensing and data processing, this paragraph describes in detail the methods used. In this study, Aluminum Nitride piezoelectric transducers generate and detect 100ns wide 1.55 GHz ultrasonic wave packets at 1 MHz repetition. The experimental setup is shown in Figures 1 and S3, with the silicon chip in contact with the sensing medium of interest. The fluid layer was a 100 *μL* thick aqueous solution of varying concentrations and ionic species. A PDMS gasket controlled the volume of this sample. First, different ionic solutions were prepared and tested with the same deionized water reference calibration of the sensing system. The reference value was set to the 0 mV reference for a deionized water backing. Thus the signal obtained from the oscilloscope following differential amplification was $V_{O}=V_{SHOS}-V_{SHOW}$. Here $V_{O}$, $V_{SHOS}$, $V_{SHOW}$ are the output voltage, the return voltage with the sample, and the return voltage with the deionized water sample. All amplifications were post-processed to be calibrated to a 3 M NaCl solution with a calibration constant to compensate for any phase error for each new acquisition. Ionic solutions were prepared using serial dilutions of a stock solution with calibrated pipettors. Sodium Chloride (NaCl), Potassium Chloride (KCl), and Calcium Chloride (CaCl_2_) preparations were made, given their biological significance in the human body as central ions used for neural, cardiac, and muscular signal conduction and of further importance in materials and environmental applications. The stock solutions were prepared with a 100 × 0.001g analytical balance with maximum tested concentrations at or under 90% solubility limit to prevent any precipitate interfering with acoustic impedance measurements. Acoustic impedance was measured across a range of concentrations from 0 mM to > 2M and high resolutions across select ranges. Five separate acquisitions per concentration were obtained, each with the average return signal from >200 consecutive reflections. Standard deviation is reported across acquisitions as this is the larger of the intra- and inter-acquisition variability and provides the more conservative measure of variability.

For the dynamic ionic content sensing for dissolution across temperatures, the following paragraphs describe the methods used. Salt dissolution is sensed with the system depicted in Figures 1 and 5, and described in the above sections. For dynamic signal change during dissolution, the sample rate should be chosen by taking into consideration the expected rate of concentration change. The signal was sampled using the oscilloscope ADC following the envelope detector and the sample-and-hold block. This method reduced the frequency content of the dissolution rate from GHz carrier frequencies with a base-band pulse repetition frequency of 1 MHz–100 kS/s sample rate, sufficient for the frequency content of salt dissolution in water.

The temperature change was induced using an annular Silicone thermal element (TSA040020eR10), with a temperature increase specified to vary approximately linearly with the voltage applied to the heater. The temperature was recorded using a K-type digital thermocouple with a temperature resolution specified at 1 degree Celsius with an error of 1.5% of the reading. This method was used to set the temperature of the solution at various values tested. To determine that water evaporation is not causing signal fluctuation, a constant concentration of 1 M NaCl was tested with multiple temperature settings during the same time course. The return signal in mV was determined to be consistent across the reading under the same sample rate of 100 kS/s for 100 seconds, ensuring that the water was not evaporating and causing a concentration spike, and only the dissolution is measured. Given that the dissolution is completed within the first 10 seconds of acquisition, 20 seconds of data are depicted to visualize the dissolution dynamics better. The reflection amplitude data was post-processed with a locally weighted linear regression (LOWESS) filter across a moving window of 1% of the data points per sample.

### Quantification and statistical analysis

All ionic content measurements for constant concentrations are reported as mean and standard deviation. The graphical depictions with error bars depict standard deviation in all cases, for both the transducer characterization and ionic content sensing applications. The mean is taken across >200 consecutive return echo amplitudes for each measurement, and 5 measurements for each reported concentration. Dynamic measurements are filtered using locally weighted filtering techniques, with details for each data acquisition reported in the method details section. Statistical testing and filtering is performed on Matlab 2021b. ANOVA with Post-Hoc Tukey HSD testing is performed for the 0 to 5 mM concentration range, and results are significant at a significance level of *α* = 0.05, the post-hoc testing suggests significance across almost all pairs of concentrations with at least p<0.05 and many pairs significant with a p<0.01, except between datapoints 1 mM and 2 mM which did not have a significant difference.

## Data Availability

• No standardized datatypes are reported in this paper. All data generated in this study is available in the figures section and will be further provided by the lead contact, Priya S. Balasubramanian (psb79@cornell.edu) upon request. • No original code is generated by this study. All processing is detailed in the STAR Methods section. • Any additional information required to reanalyze the data reported in this paper is available from the lead contact, Priya S. Balasubramanian (psb79@cornell.edu) upon request.
