# Supplementary material for: GHz ultrasonic sensor for ionic content with high sensitivity and localization
Source: iScience. 2023 May 19;26(6):106907. doi: 10.1016/j.isci.2023.106907 (PMC10250832; doi:10.1016/j.isci.2023.106907)
Supplement: Document S1. Figures S1–S3 [file mmc1.pdf]

iScience, Volume *26*

Supplemental information

GHz ultrasonic sensor for ionic content with high sensitivity and localization

Priya S. Balasubramanian and Amit Lal

Supplementary Information

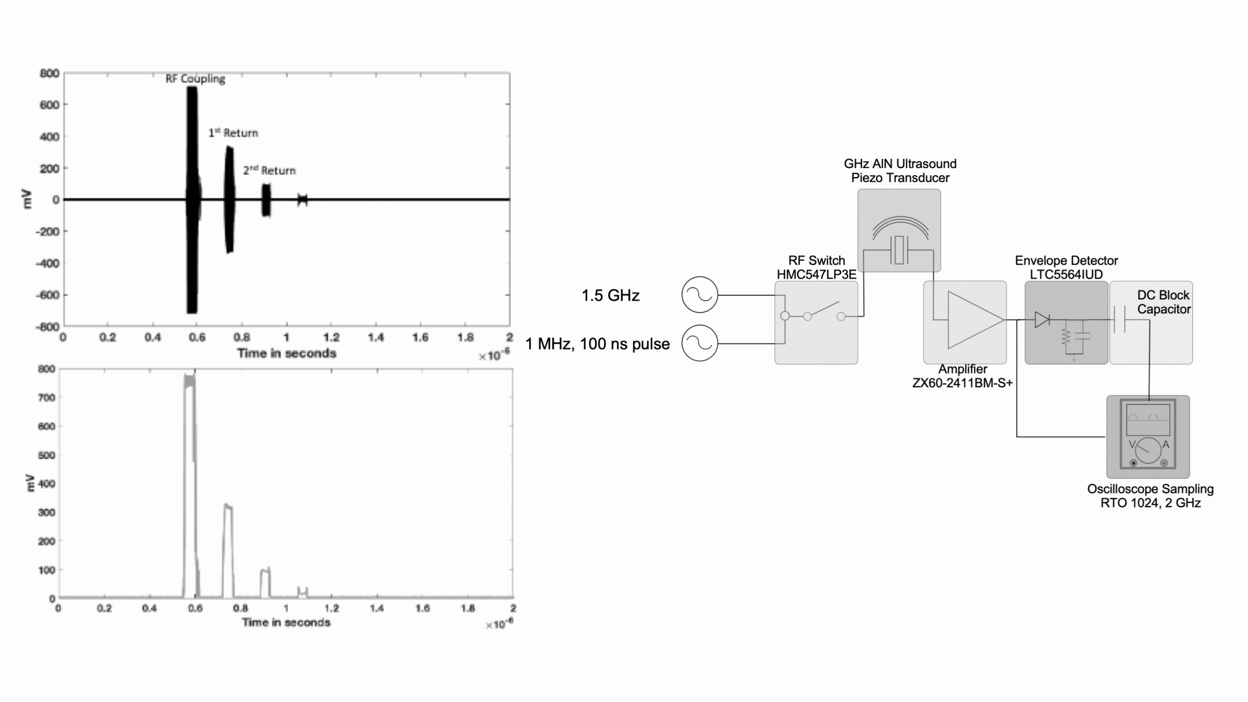

Supplemental Figure 1: Pulse Echo Return of Transducer. Related to Results. The left figure depicts the direct echo return from the transducer, with digital post-processing filter applied. The right figure depicts the analog processing to obtain these signals.

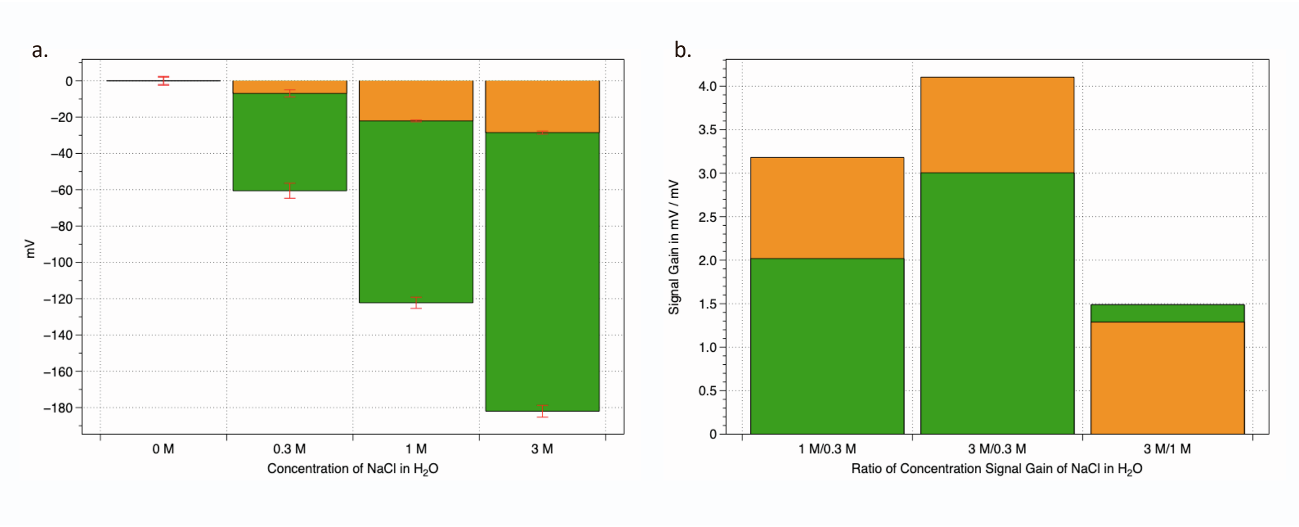

Supplemental Figure 2: First and Second Echo Comparison. Related to STAR Methods. The measurement of the RF coupled signal vs concentration for both first and second echo returns is shown to provide validity that the effect is seen and compounded in both echoes.

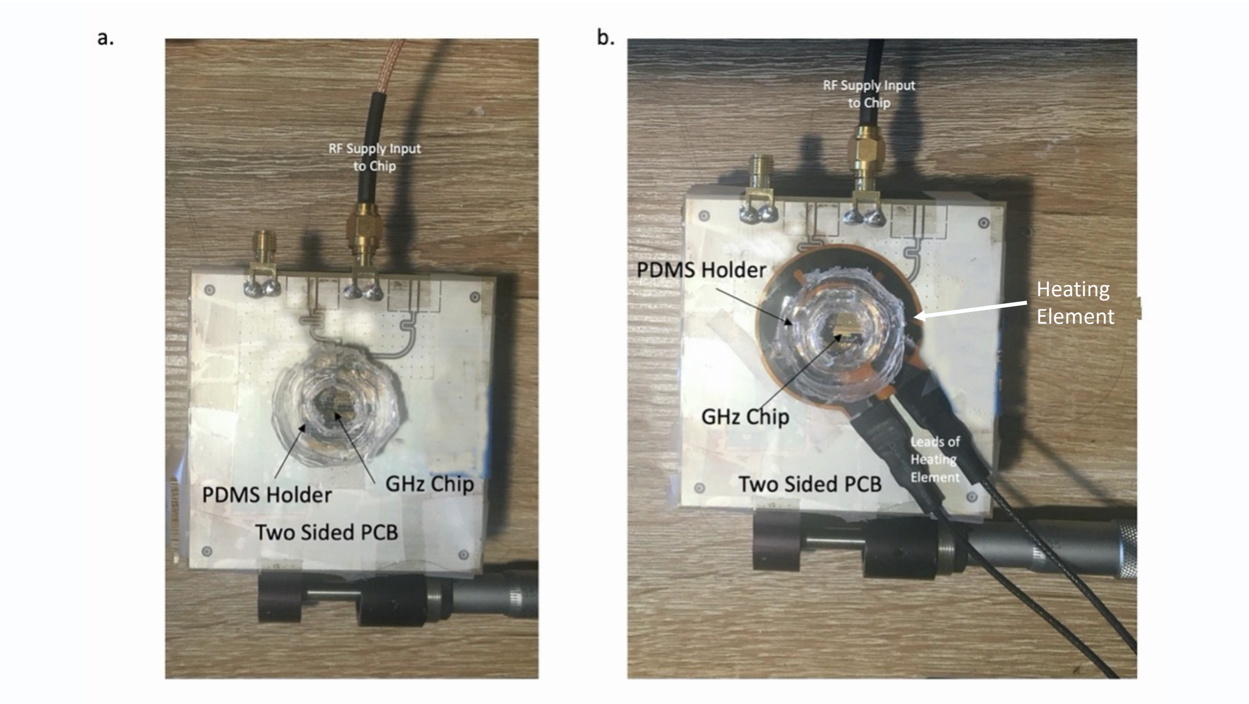

Supplemental Figure 3: Experimental Setup. Related to STAR Methods. A photograph of the experimental setup is shown, with subfigure a depicting the device for salt concentration static and dynamic measurements and subfigure b depicting the setup for thermal control of dissolution.
